# Gynaecology and general practitioner services utilisation by women in the age group 50 years and older

**DOI:** 10.25646/6808

**Published:** 2020-06-30

**Authors:** Laura Krause, Lorena Dini, Franziska Prütz

**Affiliations:** 1 Robert Koch Institute, Berlin Department of Epidemiology and Health Monitoring; 2 Charité – Universitätsmedizin Berlin Institute of General Practice

**Keywords:** UTILISATION, GYNAECOLOGY, GENERAL PRACTITIONER, GP, WOMEN, GERMANY, DEGS1

## Abstract

There are relatively few representative data on the utilisation of physician services in Germany and its influencing. Based on data from the German Health Interview and Examination Survey for Adults (DEGS1, 2008–2011), we analyse the utilisation of gynaecology and general practitioner (GP) services, with a focus on women aged 50 years and older. We compare these findings with data from the German National Health Interview and Examination Survey 1998 (GNHIES98) and, based on this and further data, discuss possible developments. Figures for seeking GP services (over 80%) are constantly high across the entire lifespan, whereas figures for gynaecology services drop with age. Around 60% of women aged 50 years and older go to a gynaecological practice at least once a year. Socioeconomic status and place of residence are important determinants for the utilisation of services. Around half of all women aged 50 years and older sought both gynaecology and GP services at least once over a one-year period. Under 10% had only been to a gynaecologist, and around one third sought GP services only. Compared to GNHIES98, figures for GP and gynaecology services were considerably higher in DEGS1, health insurance data, however, shows no increase in the use of gynaecology services between 2008 and 2018. The results highlight the need to increase awareness among GPs of the needs of middle-aged and older women for gynaecological consultation and treatment.

## 1. Introduction

Outpatient care in Germany is mainly provided by specialists in private practice [1].Generally, the use of outpatient medical services is higher in women than in men [2, 3]. These differences by sex are most pronounced at a younger age. This is very likely due to, at least in part, the utilisation of gynaecological and obstetric services [3, 4]. However, adequate gynaecological care is important at every stage in life; for middle-aged and older women the relevant issues differ from those of women still at reproductive age [5] and will be focused more on cancer screening, menopause, uterine prolapse and incontinence [6–8]. In particular, the importance of cancer screening increases: while a majority of women undergo mammography and cervical cancer screening, uptake still decreases with age [9]. A Focus article in this issue of the Journal of Health Monitoring analyses the Reasons for women aged 50 years and older to seek gynaecological advice and treatment.

**DEGS1**

**Data holder:** Robert Koch Institute

**Objectives:** To provide reliable information about the population’s health status, health-related behaviour and health care in Germany including analysis of temporal developments and trends.

**Survey method:** Questionnaires, physical examinations and tests, a physician interview, a medication interview and laboratory investigations (blood and urine sample).

**Population:** German resident population, aged 18 and above

**Sampling:** Registry office sample; randomly selected individuals from 180 communities in Germany were invited to participate (120 original sample points of the German National Health Interview and Examination Survey 1998 and 60 new sample points).

**Participants:** N=8,151 (4,283 women; 3,868 men). The sample included persons who were newly recruited and those who had already participated in the German National Health Interview and Examination Survey 1998 (mixed design).

**Response rate:** 62% among revisiting participants and 42% first time participants

**Survey period:** 2008 to 2011

**Data protection:** DEGS1 is subject to strict compliance with the data protection regulations of the Federal Data Protection Act and has been approved by the Federal Commissioner for Data Protection and Freedom of Information in Germany. Charité – Universitätsmedizin Berlin’s ethics committee assessed the ethics of the DEGS1 study and provided its approval (No.EA2/047/08). Participation in DEGS1 was voluntary. The participants were informed about the aims and contents of the studies and about data protection. Informed consent was obtained in writing.

More information in German is available at www.degs-studie.de

Socioeconomic status (SES) and place of residence are important determinants for the utilisation of medical care [2, 3, 10–15]. People in the low SES group more frequently seek general practitioner (GP) services, while those in the high SES group tend to visit medical specialists more often. People in cities see specialist physicians more often than those in rural regions, and people in rural regions more often GPs [2, 12].

Regional differences also exist regarding the availability of outpatient medical care [1]. The concentration of GPs and medical specialists is greater for urban than for rural areas. In Germany, the Associations of Statutory Health Insurance Physicians (KVen) have to ensure that everybody has access to adequate levels of health care services in close proximity to where they live [16]. Need related planning is used as an instrument to categorise types of doctors into different levels of care (for example GP care, general medical specialist care) with planning regions of different sizes. GP services are planned at the smallest scale. For general medical specialist care, the level that includes gynaecologists, districts and district free towns are the basic unit of planning, taking surrounding areas into account too. Need related planning can be adapted according to regional conditions, for example, with regard to demographic factors. Regions with a higher proportion of older women, for example, are assumed to require fewer gynaecologists [16]. In 2019, in Oberspreewald-Lausitz, a district in the state of Brandenburg with a high proportion of women aged 50 years and older (58%), for example, there were nine gynaecologists per 100,000 people [17, 18]. In the district free town of Potsdam, where the proportion of women aged 50 years and older is smaller (41%), there were 24.5 gynaecologists per 100,000 people.

Medical needs and the increasing number of older women make it extremely important to ensure adequate gynaecological care also for this population group. The impact of these demographic developments is twofold – both the population and doctors are ageing [19]. The effect is particularly notorious in rural regions, where ever fewer doctors attend to the needs of a growing number of older people. A Fact Sheet in this issue of the Journal of Health Monitoring describes the demographic developments for women aged 50 years and older in Berlin, Brandenburg and Mecklenburg-Western Pomerania.

Funded by the Innovation Fund of the Federal Joint Committee (G-BA), the project ‘Frauen 5.0’ (Regionale Versorgung von Frauen über 49 Jahre durch Fachärztinnen und Fachärzte für Gynäkologie und für Allgemeinmedizin [20]) has therefore assessed gynaecology and GP services for women aged 50 years and older in the north east region (Berlin, Brandenburg and Mecklenburg-Western Pomerania). The project aimed to gain an overview of actual levels of care provided, and, based on this, develop concepts for adequate outpatient gynaecology services to this population group.

This overview included a general analysis of the utilisation of gynaecology and GP services by women focused on the age group 50 years and older, which is in the centre of this paper. Gynaecology services for this group are rarely the focus of such analyses. As we do not have any more up-to-date population representative data on the utilisation of specialist medical services from Robert Koch Institute (RKI) health monitoring, we have opted to take the following approach: based on data from the German Health Interview and Examination Survey for Adults (DEGS1, 2008–2011), we illustrate the utilisation of gynaecology and GP services by women throughout their lifetime. For women aged 50 years and older, we will also analyse the utilisation according to SES and place of residence, as well as the correlations between the use of gynaecology and GP services. To evaluate trends, we will discuss utilisation data from DEGS1 by comparing it to the figures reported in the precursor German National Health Interview and Examination Survey 1998 (GNHIES98). Possible current developments of utilisation will be discussed based on health insurance provider and cancer screening uptake data.

## 2. Methodology

### 2.1 Sample design and study implementation

With DEGS1 the RKI has collected representative health data for the 18- to 79-year old German adult population. The study programme included interviews, physical examinations and tests. 8,151 people took part. A description of the concept and design of DEGS1 has previously been provided [21, 22].

In addition to health status and health behaviour, a focus was on the utilisation of health services. In this category, DEGS1 asked about the use of medical specialists from different disciplines. Participants were asked how often during the last twelve months they had seen a resident physician (12-month prevalence of utilisation). They could also report how many times they had contacted physicians (12-month prevalence of contact frequency). Gynaecology and GP services were among the 13 specialisations participants could choose from [2].

In addition to sex and age, DEGS1 also surveyed the sociodemographic variables SES and place of residence. SES was determined by applying a multidimensional index comprising data on education and occupational training, occupational status and net household income (needs-weighted). Based on this, participants were subdivided into low, medium and high SES groups [23]. As regards place of residence, rural and urban environments are divided into four subcategories: rural (<5,000 inhabitants), small towns (5,000–≤20,000 inhabitants), medium-sized towns (20,000–≤100,000 inhabitants) and large cities (>100,000 inhabitants) [2].

### 2.2 Statistical methods

Based on DEGS1 data, the 12-month prevalence for the utilisation of gynaecology and GP services by 18- to 79-year-old women (n=4,283) was analysed over the course of age. Then, the utilisation of gynaecology and GP services by women in the age group 50 years and older was mapped, and differences by SES and place of residence were reported (n=2,287). Regarding indicators on utilisation, prevalences and the results of multivariate binary logistic regression were calculated, as were average values and linear regression results for indicators of contact frequency. Regression analysis were adjusted for age (categorical), SES and place of residence. A statistically significant difference between groups is assumed for corresponding p-values below 0.05. Cross table analysis between the uptake of gynaecology and GP services were then conducted in a final step and tested for correlations between the two variables by applying Pearson chi square tests.

DEGS1 calculations were carried out using a weighting factor that corrects deviations within the sample from the population structure (as at 31 December 2010) with regard to age, sex, region, German citizenship, district type and education [21]. All analyses were conducted with Stata 15.1 (Stata Corp., College Station, TX, USA, 2017) using the DEGS1 data set (Version 18). Stata survey commands were used in all analyses to account for the clustering of participants at examination locations, weighting was used in the calculation of confidence intervals and p-values [24].

## 3. Results

**Figure 1:**
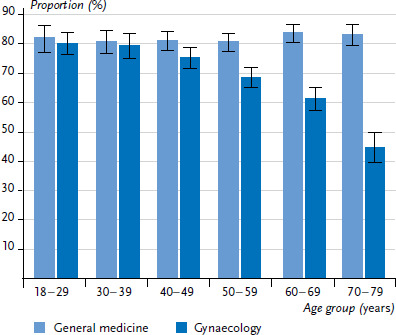
12-month prevalence of the utilisation of gynaecology and general practitioner services by 18- to 79-year-old women over time (n=4,238) Source: Modified according to Rattay et al. 2013 [2]

Around 80% of women across all age groups take up GP services within one year (Figure 1). For the use of gynaecology services, in contrast, 12-month prevalence decreases with age, in particular for older age groups: while around three quarters of 40- to 49-year-old women visited a gynaecology practice during the 12-month period before the survey (75.4%), in the 50- to 59-year-old age group this figure drops to just over two thirds (68.7%). Over the entire lifespan, the 12-month prevalence for the utilisation of gynaecology services almost halves, from 80.4% to 44.2% for 18- to 29-year-old and 70- to 79-year-old women, respectively.

59.4% of the 50- to 79-year-old women have visited a gynaecologist at least once within the past year. On average, 1.5 contacts were made. 82.6% of women of this age group consulted a GP at least once a year, with an average 4.8 contacts made per year.

Table 1 shows utilisation for 50- to 79-year-old women according to SES: women in the low SES group show lower annual utilisation of gynaecology services than women in higher status groups. In contrast, women from the high SES group most seldom see a GP. Socioeconomic differences are also evident for the 12-month prevalence of contact frequency: women in the low and medium SES groups with 5.5 and 4.8 contacts respectively, saw GPs more often than women with high SES (3.6 contacts).

For the utilisation of GP services by 50- to 79-year-old women, we can see differences in relation to place of residence (Table 1) – figures are lower for women living in large cities than for those from rural areas. Overall, the statistical probability that a woman will have had at least one appointment with a GP during the last year is 2.5 times higher for women living in rural areas. While we found no differences in relation to place of residence for the 12-month prevalence of gynaecology services utilsation, they were found for contact frequency: whereas women in rural areas on average visited a gynaecologist 1.3 times per year, the figure was 1.8 for women in large cities.

Cross-table analysis reveals a correlation between the 12-month prevalence for the utilisation of gynaecology and GP services (p<0.001): around half of all 50- to 79-year-old women (50.9%) had at least one annual visit at both a gynaecologist and GP. 31.7% of women of this age group had only seen a GP. 8.5% of 50- to 79-year-old women had gone to a gynaecologist but not to a GP during the last 12 months. 8.9% reported that they had neither have visited a gynaecologist nor a GP.

## 4. Discussion

Whereas the 12-month prevalence for the utilisation of GP services is constantly high (over 80%) across all age groups [2], the 12-month prevalence for gynaecology services utilisation decreases with age. Only around 60% of 50- to 79-year-old women have visited a gynaecologist at least once per year (utilisation across all age groups is 69.6%) [2]. Analyses of the Study of Health in Pomerania (SHIP) for the region Western Pomerania provide similar results [25]: utilisation of gynaecology services decreases from 86.3% to 36.6% for 20- to 29-year olds and women aged 70 years and older, respectively. In the 50- to 79-year age group, 71.1% of women see a gynaecologist (utilisation across all groups: 69.8%).

SES differences are a relevant factor. For women in the age group 50 years and older with low SES, figures for the utilisation of GP services are higher, whereas they are lower for gynaecology services. These findings are in line with the studies referenced at the beginning on social inequalities in the utilisation of GP and specialist medical services by the German population [2, 3, 10–15], as well as by studies on the age group 50 years and older [26–29]. A further important point in this regard are studies that show that the utilisation of services with more prevention-oriented types of medical specialists such as dermatologists, gynaecologists or dentists is lower among those with low SES and that people in this group also less frequently use prevention-oriented services as early detection and screening examinations [2, 30].

Differences in the utilisation of GP and specialist medical services by the German population found between urban and rural areas [2, 12] are in part reflected in the group of women aged 50 years and older: compared to women in large cities, women in rural areas more frequently seek GP services, with no differences between urban and rural areas found for the utilisation of gynaecology services. The study by Stentzel et al. [31] confirms this finding, showing that even when gynaecologists are hard to reach, this does not impact the use of services. According to DEGS1 data, women in rural regions report having made less contact with gynaecology practices within the last year than women in large cities. These results call for further analysis of differences in the utilisation of gynaecology services also with regard to the actually available services.

From GNHIES98 to DEGS1, the use of GP services by women has increased considerably across all age groups [2]. When observed as a whole, utilisation of GP services has increased by 10% between the two surveys, from 70.9% to 79.4%. An increase in utilisation has also been registered for gynaecology services. This increase is mainly owing to older age groups: for the 60- to 69-year-old age group, use of gynaecology services has increased by 18%, from 44% in GNHIES98 to 62% in DEGS1, in the 70- to 79-year-old age group there has even been a 24% increase from 21% to 45%, respectively [2]. Health insurance claims data can help estimate whether the utilisation of services has continued to increase since. Every year, the BARMER health insurance provides a report which analyses the utilisation of outpatient services. In 2008, 26.6% of the population took up gynaecology services [32], in 2018 25.0 % [33]. In the case of utilisation, therefore, no important developments were found, these figures were, however, not differentiated by age groups. Due to the different data basis and methodology (such as including men in the calculations) a comparison with the DEGS1 data is not possible. Data on the utilisation of cancer screening examinations provides further indications of more recent developments. The current wave of the German Health Update (GEDA 2014/2015-EHIS) study of the Robert Koch Institute reported that 53.1% of women aged 20 years and older had a screening examination for cervical cancer (pap smear) during the last twelve months. The proportion was highest for 30- to 34-year-old women (67.9%). Of 60- to 64-year-olds, fewer than half (49.0%) had this examination, in the age group 70 years and older less than a third (29.7%) [9]. Analyses of data from the German Mammography Screening Programme show that 49% of women who were invited to the Mammography Screening Programme did take part in 2017. From 2008 to 2013, participation rates by invited women increased, but have been decreasing since 2014. An increase in participation rates was only observed for women who had already previously taken part in screening and had been invited again [34]. Mammography screening does not take place in gynaecology practices. However, this could be an opportunity to seek consultation in a gynaecology practice. Further reasons for consultation and treatment that play a role for women aged 50 years and older beyond cancer early detection are discussed in a second Focus article in this issue of the Journal of Health Monitoring.

Surveys of utilisation through population-based studies such as RKI health surveys have certain limitations that are important to discuss. Self-reported data on utilisation of medical services can under-report utilisation or contain errors, for example due to recall bias, or, because very old or sick people are not included in the survey [35, 36]. Underestimating the number of appointments appears to occur particularly often at an older age [37], however, more with regard to the number of contacts made than whether medical services had been sought at all. The probability of recall bias also increases, when periods of over 12 months are considered [38]. DEGS1 data, unlike health insurance claims data, provides findings on utilisation of services independently of type of health insurance [36].

These results provide no direct conclusions on the degree by which actual treatment needs are met and on the quality of services, yet they do highlight the need to analyse the reasons why women in the age group 50 years and older decide not to seek gynaecology services. It is possible that women link gynaecology services to reproductive health and therefore consider appointments as not or no longer necessary following a certain age, or that women consider examinations as being (too) unpleasant. Analysis from the project ‘Frauen 5.0’ indicate that personal barriers (age, shame or being afraid of a gynaecological examination), as well as the general framework of care provision (such as long waiting times, long distances) are potential reasons for not seeking an appointment [39, 40]. It could however also be that the increase in utilisation of gynaecology services evident for the years from 1998 and 2008–2011 has continued and that women in younger age cohorts will seek gynaecology services more frequently. Further – also qualitative – studies should therefore be conducted in the future and focus on the use of services by middle-aged and older women. The Fact Sheet Barriers for women aged 50 years and older to accessing health care in Germany describes selected access barriers to outpatient care in greater detail.

Strenghtening health literacy can lead to a need-oriented utilisation of gynaecology services, for example by providing information on gynaecological diseases or cancer screening [39]. DEGS1 data shows another possible option: around one third of women in the age group 50 years and older has only consulted GP services. Accordingly, GPs could be trained more to meet the gynaecological consultation and treatment needs of middle-aged and older women. This applies in particular to cancer screening [41]. Based on actual care provision, one aim of the project ‘Frauen 5.0’ is to develop a model for regional outpatient care which – also through interprofessional co-operation – helps meet the actual treatment needs of women aged 50 years and older [39].

## Key statements

- Utilisation of gynaecology services by women decreases with age, figures for GP services are constantly high across all age groups.
- A considerable decrease in the utilisation of gynaecology services is observed for women in the 50- to 59-year age group.
- Around one third of women aged 50 years and older had an appointment with a GP only and not with a gynaecologist.
- Utilisation of GP and gynaecology services between 1998 and 2008–2011 by women aged 50 years and older has increased considerably.

## Figures and Tables

**Table 1:** 12-month prevalence for the utilisation of gynaecology and general practitioner services by 50- to 79-year-old women and results of multivariate binary logistic regression analysis (odds ratios) by socioeconomic status and place of residence (n=2,287) Source: DEGS1 (2008–2011)

	Utilisation of gynaecology services					Utilisation of general practitioner services				
	%	(95% CI)	OR	(95% CI)	p-value	%	(95% CI)	OR	(95% CI)	p-value
**Socioeconomic status**										
Low	47.1	(41.1–53.1)	0.56	(0.37–0.84)	0.005	81.8	(75.8–86.6)	1.22	(0.82–1.82)	0.317
Medium	61.8	(58.5–65.0)	0.88	(0.65–1.20)	0.428	84.7	(82.4–86.8)	1.63	(1.19–2.23)	0.003
High	68.0	(62.3–73.2)	Ref.	Ref.	–	75.7	(70.4–80.3)	Ref.	Ref.	–
**Place of residence**										
Rural	55.6	(49.7–61.3)	0.79	(0.57–1.11)	0.175	89.6	(85.9–92.4)	2.46	(1.55–3.91)	< 0.001
Small towns	56.4	(52.0–60.7)	0.78	(0.59–1.03)	0.076	82.4	(77.7–86.2)	1.29	(0.87–1.92)	0.208
Medium-sized towns	60.6	(55.8–65.2)	0.87	(0.66–1.15)	0.327	83.1	(80.0–85.8)	1.36	(0.96–1.91)	0.082
Large cities	62.8	(58.0–67.3)	Ref.	Ref.	–	78.3	(73.3–82.6)	Ref.	Ref.	–

CI=Confidence intervals, OR=Odds ratio, Ref.=Reference group
